# Repurposing the Public BraTS Dataset for Postoperative Brain Tumour Treatment Response Monitoring

**DOI:** 10.3390/tomography10090105

**Published:** 2024-09-01

**Authors:** Peter Jagd Sørensen, Claes Nøhr Ladefoged, Vibeke Andrée Larsen, Flemming Littrup Andersen, Michael Bachmann Nielsen, Hans Skovgaard Poulsen, Jonathan Frederik Carlsen, Adam Espe Hansen

**Affiliations:** 1 Department of Radiology, Centre of Diagnostic Investigation, Copenhagen University Hospital—Rigshospitalet, 2100 Copenhagen, Denmark; vibeke.andree.larsen@regionh.dk (V.A.L.); michael.bachmann.nielsen@regionh.dk (M.B.N.); jonathan.frederik.carlsen@regionh.dk (J.F.C.); adam.espe.hansen@regionh.dk (A.E.H.); 2 Department of Clinical Medicine, University of Copenhagen, 2100 Copenhagen, Denmark; flemming.andersen@regionh.dk; 3 The DCCC Brain Tumor Center, 2100 Copenhagen, Denmark; hans.skovgaard.poulsen@regionh.dk; 4 Department of Clinical Physiology and Nuclear Medicine, Centre of Diagnostic Investigation, Copenhagen University Hospital—Rigshospitalet, 2100 Copenhagen, Denmark; claes.noehr.ladefoged@regionh.dk; 5 Department of Applied Mathematics and Computer Science, Technical University of Denmark, 2800 Lyngby, Denmark

**Keywords:** brain tumour segmentation, treatment monitoring, postoperative, annotation protocol, automatic, deep learning algorithm, magnetic resonance imaging, MRI, Brain Tumor Segmentation Challenge, BraTS

## Abstract

The Brain Tumor Segmentation (BraTS) Challenge has been a main driver of the development of deep learning (DL) algorithms and provides by far the largest publicly available expert-annotated brain tumour dataset but contains solely preoperative examinations. The aim of our study was to facilitate the use of the BraTS dataset for training DL brain tumour segmentation algorithms for a postoperative setting. To this end, we introduced an automatic conversion of the three-label BraTS annotation protocol to a two-label annotation protocol suitable for postoperative brain tumour segmentation. To assess the viability of the label conversion, we trained a DL algorithm using both the three-label and the two-label annotation protocols. We assessed the models pre- and postoperatively and compared the performance with a state-of-the-art DL method. The DL algorithm trained using the BraTS three-label annotation misclassified parts of 10 out of 41 fluid-filled resection cavities in 72 postoperative glioblastoma MRIs, whereas the two-label model showed no such inaccuracies. The tumour segmentation performance of the two-label model both pre- and postoperatively was comparable to that of a state-of-the-art algorithm for tumour volumes larger than 1 cm^3^. Our study enables using the BraTS dataset as a basis for the training of DL algorithms for postoperative tumour segmentation.

## 1. Introduction

The measurement of the volume of brain tumours has the potential to be an important biomarker for both the routine response assessment of cancer patients as well as for clinical trials. In recent years, there have been substantial efforts towards automating the segmentation of brain tumours on magnetic resonance imaging (MRI), especially by deep learning (DL) algorithms [1,2]. Among these, the nnU-Net framework [3] has distinguished itself as a self-configuring method that adapts to varying segmentation tasks, demonstrating leading performance in multiple benchmark challenges, including the Brain Tumor Segmentation (BraTS) Challenge [4,5,6]. Today, DL brain tumour segmentation algorithms are achieving over 90% agreement with imaging experts [5,7]. The BraTS Challenge has been the main driver in the development of the field, providing by far the largest publicly available training dataset comprising 1251 MRI examinations of glioma patients with expert reference annotations [8]. However, the BraTS dataset derives exclusively from preoperative MRI examinations.

The majority of brain tumour patients undergo surgery as part of the initial treatment and are subsequently monitored by MRI [9,10,11]. Surgical procedures can result in cavities, tissue scarring and haematomas, all of which introduce image features typically absent preoperatively. The current BraTS annotation protocol is tailored for preoperative MRI and does not accommodate these postoperative features. Therefore, DL algorithms trained only by use of the BraTS dataset may be suboptimal for response assessment. The ideal source for training a DL algorithm for treatment response monitoring would be a comprehensive, expert-annotated dataset including postoperative MRIs. To the best of our knowledge, no suitable expert-annotated postoperative datasets are publicly available, hampering the development of DL algorithms for treatment response monitoring. HD-GLIO, an nnU-Net model trained on postoperative but non-public data, indicates the feasibility and potential of such endeavours [12,13,14], although its application in routine response-monitoring settings may be challenged when small contrast-enhancing tumours are prevalent [15].

The aim of this study is to aid the development of DL algorithms for postoperative brain tumour treatment response monitoring by facilitating the use of the preoperative BraTS dataset. We hypothesise that a state-of-the-art DL algorithm, when trained on the BraTS dataset with its current annotations, is unsuitable for postoperative segmentation due to the potential for misclassifying resection cavities as tumour regions. To remedy this shortcoming, we introduce a revised annotation protocol that aligns with postoperative tumour segmentation. Accordingly, we further hypothesise that when the DL algorithm is retrained using our proposed annotation protocol on the same MRI data, it will accurately exclude resection cavities from tumour volumes in postoperative cases, and thus significantly improve its usability in the postoperative setting. By validating these hypotheses, we seek to equip a broad community of researchers and clinicians with a large body of training data to develop algorithms tailored for postoperative brain tumour response assessment.

## 2. Materials and Methods

We trained a deep learning brain tumour segmentation algorithm using the BraTS dataset and annotation protocol as well as our proposed protocol. The datasets, annotation protocols and evaluations are described below.

### 2.1. Preoperative Public Dataset

We utilised the most recent BraTS adult glioma training dataset curated for the RSNA-ASNR-MICCAI BraTS 2021 Challenge [4,6,8,16] for training. The BraTS 2021 training dataset has been sourced from multiple institutions and comprises 1251 independent preoperative patient scans, each confirmed with a subsequent pathological diagnosis of glioma. Each scan encompasses four MRI sequences: T1-weighted before contrast infusion (T1), T1-weighted after contrast infusion (T1Gd), T2-weighted and fluid-attenuated inversion-recovery (FLAIR). These sequences have been preprocessed as described in [17,18,19], which includes co-registration, resampling to voxel size of 1 mm^3^ and skull-stripping. For our study, we selected a subset consisting of the last 1000 out of the 1251 scans to train the DL algorithms. We used the remaining first 251 scans as a test set to evaluate preoperative tumour segmentation performance.

### 2.2. Postoperative Public Test Dataset

For testing the performance of the algorithms in a postoperative setting, we employed the publicly available Lumiere dataset [20]. The dataset encompasses scans of 91 patients with a confirmed diagnosis of glioblastoma. These patients underwent preoperative MRI between 2008 and 2013, received surgical resection treatment, underwent chemotherapy and had routine follow-up MRIs. The scans include T1, T1Gd, T2 and FLAIR sequences. In the Lumiere dataset, a version of these images has been preprocessed with HD-GLIO AUTO [12,21,22], complete with co-registration, resampling and skull-stripping. For our study, from the preprocessed dataset, we chose the earliest follow-up MRI after week 11 for each of the 72 patients where that particular scan was complete with all four sequences.

### 2.3. BraTS Three-Label Annotations

Historically, the BraTS Challenges have implemented an annotation protocol that subsegmented tumours into four distinct regions based on specific radiological observations. They include the active Gadolinium-enhancing tumour (AT), necrosis (NCR), non-enhancing tumour core (NET) and surrounding oedematous or infiltrated tissue (ED). In 2017, the protocol was simplified by merging NCR and NET into a single label (NCR+NET) [5]. Hence, NCR+NET encompasses both necrosis and non-enhancing tumour core. Recently, AT has been renamed to ET (enhancing tumour), and NCR+NET to just NCR [8]. For clarity, in this study, we will, however, refer to BraTS annotations by their original names, AT and NCR+NET as well as ED.

### 2.4. Proposed Two-Label Annotation Protocol

Our proposed annotation protocol adapts and expands the BraTS annotation protocol [5,8] to make it suitable for both pre- and postoperative settings. We employ two labels: the contrast-enhancing tumour (CE) and the non-enhancing hyperintense T2-FLAIR signal abnormalities (NEs) inspired by [12], with more detailed directions given below. The annotation protocol is in accordance with the relevant VASARI MR feature definitions [23] and with adaptations for postoperative compatibility. Specifically, necrotic and cystic regions are not incorporated in either label. They do not represent viable tumour tissue and may share image features with resection cavities.

The CE label is defined similarly to the BraTS AT label but with the morphological changes from surgery taken into consideration. If a region is hyperintense on T1, it should not be categorised under the CE label unless it distinctly enhances on T1Gd and exhibits a pattern different from that observed on T1. Enhancements on T1Gd with a simple, thin geometric configuration along the resection walls are interpreted as benign changes resulting from surgery and are thus excluded from the CE label. On the other hand, locally thickened or nodular enhancements seen along the resection walls should be included in the CE label. Dural thickening and leptomeningeal enhancement on T1Gd, much more often a result of surgery than glioma infiltration, should not fall under the CE label. We recognise that distinguishing between the two can occasionally pose challenges. Closely configured, sporadic enhancements in areas with high T2/FLAIR-signal are viewed as indicators of angiogenesis due to tumour growth and should thus be included in the CE label.

The NE label closely resembles a combination of the original BraTS NET and ED labels but comes with additional clarifications. T2/FLAIR hyperintensity, when adjacent to a resection cavity, indicating the presence of a previous tumour, should be equated with peritumoural oedema. As such, T2/FLAIR hyperintensity connected with a resection cavity should be allocated to the NE label. However, regions showing hyperintensity on T2/FLAIR, even if adjacent to an enhancing tumour, non-enhancing tumour core or a resection cavity, should be excluded from the NE label if they manifest a configuration mirrored contralaterally or if they evidently resemble typical periventricular oedema, a common treatment-induced feature, or white matter hyperintensities of probable vascular origin. We acknowledge the occasional difficulty in precisely determining the boundary between peritumoural oedema and unrelated periventricular oedema. Necrosis and cysts should not be allocated to the NE label.

### 2.5. HD-GLIO

HD-GLIO is a freely accessible DL brain tumour segmentation algorithm designed for postoperative treatment response assessment [12]. HD-GLIO differentiates between contrast-enhancing (CE_HD-GLIO_) tumours and non-enhancing hyperintense T2/FLAIR signal abnormalities (NE_HD-GLIO_), while excluding necrosis, obvious leucoaraiosis (white matter hyperintensities of probable vascular origin) and resection cavities. The algorithm employs the nnU-Net framework [21] and was trained on a non-public dataset comprising 455 independent pre- or postoperative adult glioma MRIs. It has been validated on two longitudinal datasets: a local set of 239 MRIs from 40 glioma patients and a large multi-centre set of 2034 MRIs from 532 glioblastoma patients in the EORTC-26101 cohort. In our study, HD-GLIO served two purposes: (1) it provided a reliable whole-tumour segmentation, excluding necrosis; and (2) it acted as a comparative benchmark for evaluating the performance of our algorithm. We used HD-GLIO version 1.5 [24].

### 2.6. Conversion of BraTS Annotations

To harmonise the BraTS training data annotations with our newly proposed annotation protocol, we devised an algorithm for the systematic conversion of the BraTS segmentation masks. This conversion process is outlined below:

1. With HD-GLIO, obtain a whole tumour segmentation that excludes necrosis, WT_HD-GLIO_ = CE_HD-GLIO_ + NE_HD-GLIO_.
2. Assign the NCR+NET segmentations within WT_HD-GLIO_ to the NE label. This is the only step where the HD-GLIO segmentation is used.
3. Add the ED segmentations to the NE label.
4. Remove voxels in the NE label that are entirely encapsulated by AT segmentations, as these typify necrosis.
5. Remove voxel clusters in the NE label that are less than 50 mm^3^ to eliminate potentially isolated traces of what was previously NCR segmentations.
6. Add again the ED segmentations to the NE label to ensure complete incorporation of the ED segmentations.
7. Assign the AT segmentation to the CE label.

**Figure 1:**
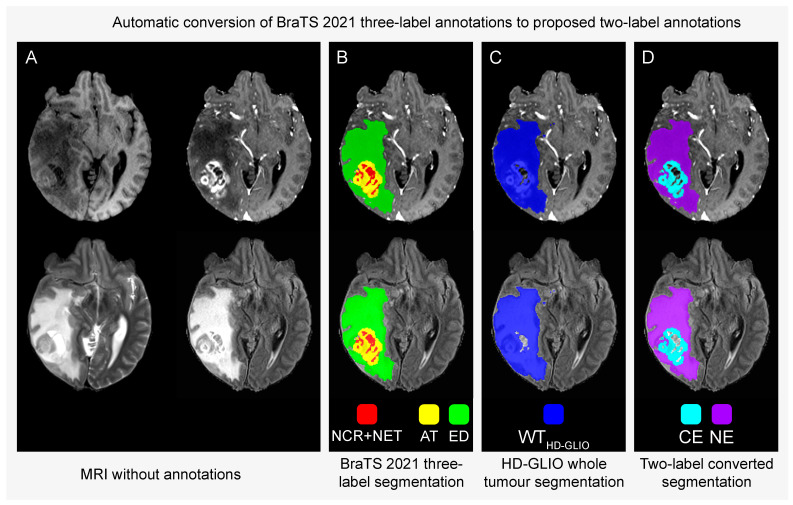
Conversion of the BraTS 2021 three-label segmentation masks for harmonisation with the proposed two-label annotation protocol. **Panel A**: Anatomical sequences without annotations. T1-weighted (T1) (top left), T1-weighted after contrast infusion (T1Gd) (top right), T2-weighted (bottom left) and fluid-attenuated-inversion-recovery (FLAIR) (bottom right). **Panels B**–**D**: annotations overlaid on T1Gd (top) and FLAIR (bottom). NCR+NET = necrosis, cysts and non-enhancing tumour core; AT = active contrast-enhancing tumour; ED = oedema and infiltrated tissue; WT_HD-GLIO_ = automatic whole-tumour segmentation by HD-GLIO; CE = contrast-enhancing tumour; NE = non-enhancing hyperintense T2/FLAIR signal abnormalities. In essence, the conversion process labels the combination of the NCR+NET mask inside the WT_HD-GLIO_ region and the ED mask as NE, thereby removing necrosis and cysts while retaining non-enhancing tumour core. AT is relabelled as CE without change. Only the regions inside the NCR+NET segmentations (red) are affected by the conversion.

The result of the conversion is that the CE label is identical to the original AT label, and the NE label includes the full original ED label as well as the estimated NET regions of the original NCR+NET label. Thus, the conversion process exclusively removes voxels inside the NCR+NET regions of the BraTS segmentations. Assuming that the original masks adhere to the BraTS Challenge’s annotation protocol, our process is designed to remove necrosis and cysts. Figure 1 illustrates the conversion process in one example, and Appendix A provides a schematic illustration of the process. The label conversion script is freely available on GitHub [25].

### 2.7. Training of Deep Learning Algorithm

We utilised the nnU-Net architecture with standard settings in the ‘3d_fullres’ configuration to train our deep learning model on two distinct annotation sets. The first set was the expert-annotations from the BraTS 2021 dataset, adhering to the BraTS three-label annotation protocol. The second set, based on our newly proposed two-label protocol, was derived by conversion from the initial set as detailed in the previous section. Both training processes employed the same 1000 scans from the BraTS 2021 training dataset. See Appendix B for technical details.

### 2.8. Evaluation

To assess the conversion of the original three-label annotations in the BraTS dataset to our recommended two-label annotations, we conducted a subjective evaluation of every 10th case, 125 cases in total, and calculated the conversion success rate with 95% confidence interval (95% CI) using the conservative Clopper–Pearson (exact) method that does not assume a normal distribution.

We wished to examine how frequently the three-label and two-label deep learning models included parts of resection cavities in the postoperative test dataset. To this end, we employed a custom script that demarcated fluid-filled cavities in the brain, which on z-normalised (zero mean and unit variance) signal values were characterised by T1Gd < −1, FLAIR < 0 and T2 > 0 in a continuous volume of at least 2 cm^3^, and subsequently validated these delineations by manual inspection before the analysis of all relevant volumes. We report how frequently the three-label and two-label models included parts of the identified cavities, and the volume included.

To verify that training of the deep learning model using converted labels did not compromise the tumour segmentation, we evaluated the segmentation both pre- and postoperatively. For evaluation of the preoperative tumour segmentation performance of the two-label model, we used as reference the converted annotations from the scans in the BraTS dataset that were kept out of the training dataset and determined Dice similarity scores (DSCs). As a benchmark, we also applied HD-GLIO and tested if its postoperative performance was significantly different from that of our model using the Wilcoxon rank sum exact test. Bootstrapping in R version 4.4.1 was used to calculate 95% CIs [26].

For evaluation of the postoperative tumour segmentation performance of the two-label model, we manually segmented the postoperative test dataset tumours using ITK-SNAP version 4.0.0 [27,28] in line with the two-label annotation protocol. Utilising these manual expert-provided segmentations as our reference, we determined DSCs for the segmentations generated by the two-label model. Likewise, for benchmarking, we evaluated the segmentations by HD-GLIO AUTO that was provided as part of the Lumiere dataset.

## 3. Results

We automatically converted all 1251 segmentations from the BraTS 2021 training dataset from the original three-label annotations to our proposed two-label annotations. A subjective evaluation of 125 cases (every 10th case) showed, with three exceptions, consistent success in the conversion process, yielding a conversion success rate of 122/125 = 97.6% (95% CI [93.2–99.5%]). Inspection showed that the removed internal areas from the original NCR+NET labels corresponded to probable necrotic and cystic regions, provided the original annotations adhered to the BraTS annotation protocol. The cases with discrepancies were two cases with substantial NET tumours in which the WT_HD-GLIO_ left out parts of the less intense FLAIR hyperintensities, and one case where central necrosis was included in the WT_HD-GLIO_ while not completely encapsulated in AT. Thus, in two cases, NET was erroneously excluded from the resulting NE label, and in one case, NCR was erroneously included in it. Typically, in cases with large NET volumes (lower grade gliomas), there were slight variations in the external boundary between the original three-label segmentation and the converted two-label segmentation. Generally, the dissimilarities between WT_HD-GLIO_—used in step 2 of the conversion—and the original BraTS three-label segmentations were as desired within the internal probable necrotic and cystic regions and otherwise within the assessment of the lesion border.

**Figure 2:**
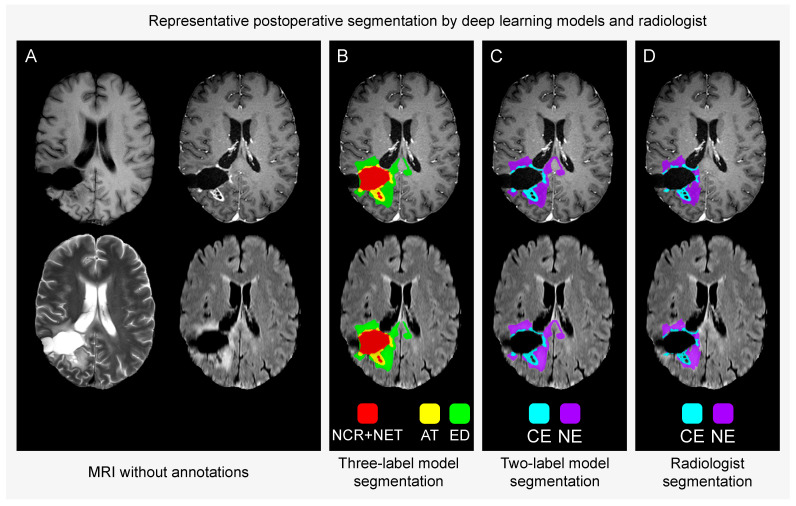
Representative case from the postoperative test set of segmentations performed by deep learning models trained utilising the BraTS three-label annotation protocol and our proposed two-label protocol and by radiologist as reference. **Panel A**: Anatomical sequences without annotations. T1 (top left), T1Gd (top right), T2 (bottom left) and FLAIR (bottom right). **Panels B**–**D**: Annotations overlaid on T1Gd (top) and FLAIR (bottom). NCR+NET = necrosis, cysts and non-enhancing tumour core; AT = active contrast-enhancing tumour; ED = oedema and infiltrated tissue; CE = contrast-enhancing tumour; NE = non-enhancing hyperintense T2/FLAIR signal abnormalities. The three-label model includes parts of the resection cavity (panel B) while the two-label model (panel C) and radiologist reference (panel D) do not.

We identified and segmented 41 fluid-filled resection cavities within the postoperative test set comprising MRI examinations from 72 patients. The deep learning model trained with expert annotations adhering to the BraTS three-label annotation protocol labelled a minimum of 1 cm^3^ in 10 of the 41 cavities. On average, the labelled volume in a cavity was 10.7 cm^3^. Conversely, the model trained with annotations adapted to our two-label protocol did not include a volume exceeding 0.1 cm^3^ in any of the cavities (and neither did HD-GLIO AUTO). Figure 2 shows a case where the three-label model included parts of the resection cavity.

**Figure 3:**
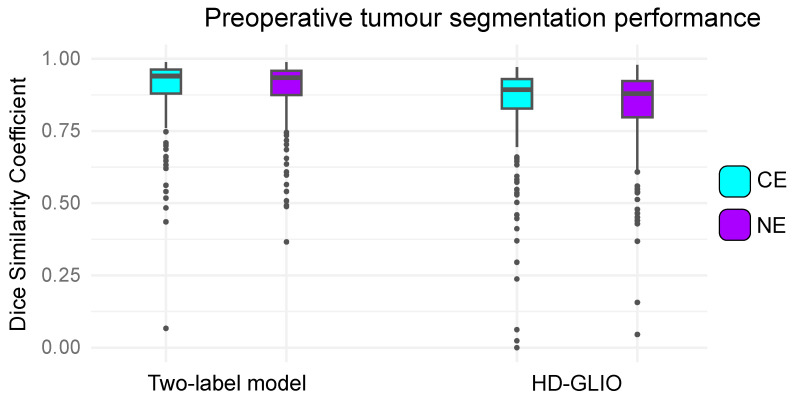
Agreement in preoperative brain tumour segmentations between deep learning models and two-label converted expert annotations across 251 cases from the BraTS 2021 training set, reserved for testing. The boxplots depict the distributions of Dice similarity coefficients (DSCs). CE = contrast-enhancing tumour; NE = non-enhancing hyperintense T2/FLAIR signal abnormalities.

For the preoperative test set, the median DSC values between the two-label model and converted reference expert segmentations were 0.94 (95% CI [0.93; 0.95]) for CE tumours and 0.94 (95% CI [0.92; 0.94]) for NEs. HD-GLIO achieved median DSC values of 0.89 (95% CI [0.88; 0.90]) for CE tumours and 0.88 (95% CI [0.87; 0.90]) for NEs. The DSC distributions are illustrated by box plots in Figure 3.

**Figure 4:**
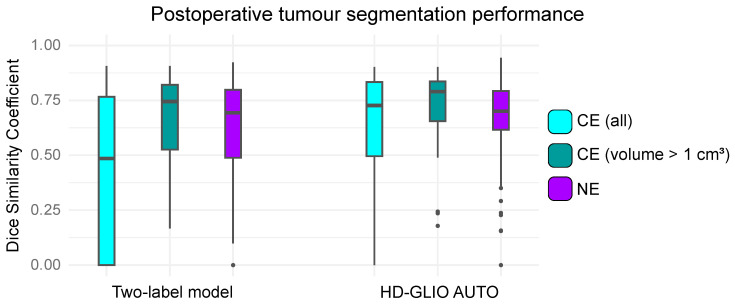
Agreement in postoperative brain tumour segmentations between deep learning models and our independent expert two-label annotations for 72 cases from the postoperative test set. The boxplots depict the distributions of Dice similarity coefficients (DSCs). CE = contrast-enhancing tumour; NE = non-enhancing hyperintense T2/FLAIR signal abnormalities; CE (volume > 1 cm^3^) = CE segmentations with volumes exceeding 1 cm^3^.

For the postoperative test set, the median DSC values between the two-label model and reference expert segmentations were 0.48 (95% CI [0.30; 0.70]) for CE tumours and 0.68 (95% CI [0.63; 0.74]) for NEs. When focusing on CE tumours larger than 1 cm^3^, the median DSC was 0.75 (95% CI [0.66; 0.83]). In contrast, the comparison of HD-GLIO’s segmentations with reference expert segmentations produced median DSC values of 0.73 (95% CI [0.63; 0.79]) for CE tumours, 0.71 (95% CI [0.68; 0.74]) for NEs and 0.79 (95% CI [0.73; 0.83] for CE tumours larger than 1 cm^3^. For CE tumours with volumes greater than 1 cm^3^ and for NEs, the two models’ performances were not significantly different (*p* = 0.40 and *p* = 0.29, respectively). The DSC distributions are illustrated by box plots in Figure 4. In 15 cases, the two-label model achieved CE DSC = 0 due to false positives. In all cases but one, the two-label model included either contrast enhancing dura above a superficial cavity or parts of an enhancing cavity wall. In one case, it included parts of plexus choroideus. In three cases, the two-label model achieved CE DSC = 0 due to false negatives. All three cases involved weakly contrast-enhancing lesions.

## 4. Discussion

In this study, we demonstrated that using the BraTS dataset, with its original three-label annotations, to train a DL algorithm for segmentation of brain tumours after surgery will result in substantial and frequent mislabelling of resection cavities. We automatically converted the three-label BraTS annotations to two-label annotations suitable for the postoperative setting and used them to train a model that excluded all cavities and showed a tumour segmentation performance comparable to that of a state-of-the-art algorithm for volumes exceeding 1 cm^3^. Hence, our study enables the use of the largest publicly available expert annotated brain tumour (BraTS) dataset for training DL algorithms aimed at treatment response monitoring.

Prior research suggesting the harmonisation of pre- and postoperative brain tumour segmentation has typically incorporated the resection cavities into the NCR+NET label [29,30,31]. This approach is useful to radiotherapy-planning for glioblastoma, where, according to clinical guidelines [32], resection cavities are incorporated into the gross tumour volume (GTV). Conversely, Kickingereder et al. [12] and Chang et al. [33] opted to label the contrast-enhancing tumour and volumes of FLAIR hyperintense signal abnormalities but not the resection cavities, thereby emphasising the active tumour growth crucial for treatment response monitoring [10,11]. This perspective is underlined by Kickingereder et al., who illustrated that the time-to-progression based on these labels served as a superior surrogate endpoint for overall survival compared to RANO assessment with two-dimensional measurements.

The studies focusing on postoperative treatment response monitoring by Chang et al. [33] and Kickingereder et al. [12] relied on newly annotated pre- and postoperative data for training DL algorithms, which are not publicly available. Our study adds to the current literature by introducing a two-label annotation protocol that adapts and expands the BraTS annotation protocol with a focus on postoperative image features. Further, the study provides a fully automatic conversion of the existing, publicly available BraTS annotations to align with our proposed two-label annotation protocol. This latter contribution positions the large BraTS training dataset as a foundational resource in the public domain for the development of deep learning algorithms designed for brain tumour treatment response monitoring.

Central to this study is the novel conversion of the expert-validated BraTS three-label segmentations to our proposed two-label format. In one of its steps, the conversion process relies on an automatic whole-tumour segmentation by HD-GLIO (WT_HD-GLIO_). Typically, the dissimilarities between WT_HD-GLIO_ and the original BraTS three-label segmentations occur as intended within the probable cystic and necrotic regions and otherwise within the assessment of the lesion border. Since only the NCR+NET label areas of the original annotations are altered in the conversion process, the cases most prone to potential conversion errors are tumours with large NET volumes (lower-grade gliomas) extending to the outer contour. In the majority of such cases, this results in only minor variations in the external NE contour. This is supported by the conversion success rate of 122/125 = 97.6% (95% CI [93.2–99.5%]). Based on our inspection, it is therefore unlikely that there are frequent issues in the conversion of the complete BraTS 2021 training dataset segmentations, and it demonstrates that the conversion remains robust against inconsistencies between WT_HD-GLIO_ and the original BraTS segmentations.

A limitation of our study is that we employed a particular U-Net model and only a single postoperative dataset. Yet, evidence suggests the issue of misclassification of resection cavities is not isolated. DeepBratumIA [34] segmentations are provided along with the Lumiere dataset [20] using a different preprocessing method involving atlas registration. Our inspections of the segmentations revealed erroneously included cavity parts [20]. Additionally, Ramesh et al. shows an example segmentation of a Swin-UNETR model [35] trained on the same BraTS data, exhibiting cavity inclusion in their published figure [31]. Our own preliminary observations with nnU-Net on a local dataset reinforce this pattern. Thus, various U-Net architectures trained on the three-label BraTS 2021 training dataset demonstrate a consistent tendency to misclassify parts of resection cavities as tumour tissue across diverse postoperative datasets and preprocessing techniques, an error which is mitigated by our two-label annotation approach.

Our two-label model shows a high rate of false positives in the postoperative setting, particularly apparent by DSC = 0 achieved in some cases with CE tumour smaller than 1 cm³. This is anticipated, given that the model was trained exclusively on preoperative MRI and thus was not exposed to the commonly occurring postoperative contrast enhancement patterns from non-tumorous tissue [36], like HD-GLIO was. Hence, some postoperative cases are necessary for training a model in this setting. However, our proposed conversion of the BraTS annotations enables the use of the large BraTS dataset for training, which may considerably reduce the need for expert-annotated postoperative data, which have limited availability [18]. The accuracy of our two-label model for larger CE tumours indicates the viability of this approach.

In assessing the automated conversion, we noted discrepancies in three out of 125 evaluated cases when compared to our expectations. Nevertheless, such discrepancies did not significantly impact the training efficacy of our two-label model, which sustained a high Dice coefficient of 0.94 for segmenting both CE tumour and NEs in the preoperative test set.

Although our two-label annotation protocol might differ slightly from the training approach used by HD-GLIO, the core criteria remain consistent: The CE label identifies contrast-enhancing tumours, while the NE label represents non-enhancing T2/FLAIR hyperintense signal abnormalities. Therefore, HD-GLIO’s segmentations are a valid benchmark for direct comparisons with our model. The relevance of our protocol lies in its derivation from and full compatibility with the BraTS preoperative protocol, which allowed for the conversion to a two-label annotation but also ensures consistent segmentation of postoperative MRIs in future research and in the development and evaluation of DL segmentation models.

In perspective, our study underscores the capability of BraTS to serve as a foundational dataset for the training of DL algorithms for postoperative tumour segmentation, pivotal for treatment response monitoring. We demonstrate that by using the converted two-label annotations, DL algorithms can accurately exclude resection cavities postoperatively while maintaining robust tumour segmentation. However, we acknowledge that training solely on the preoperative BraTS dataset may not fully address all surgically induced lesions; incorporating some postoperative data remains necessary. The key contribution is enabling the research community to effectively utilise the extensive and high-quality BraTS dataset in training DL algorithms for postoperative applications, potentially reducing the reliance on scarce postoperative training data. This contribution is particularly significant given the notable scarcity of publicly available, high-quality postoperative datasets with expert annotations. Among the few available postoperative datasets, the UPenn-GBM dataset [37] includes 60 postoperative MRIs, and the Lumiere dataset [20], which we utilised for postoperative testing, encompasses longitudinally collected MRIs from 91 patients. Unfortunately, neither dataset offers expert annotations. To our knowledge, no other public datasets currently meet the requisite standards of quality and expert annotation.

With our work, the accessibility of adapted annotations for public use paves the way for significant advancements in research. Future studies could validate the potential of combining the BraTS dataset with a carefully selected or limited set of annotated postoperative MRI cases. This combination would ideally leverage datasets from the public domain, such as the Lumiere dataset [18], to efficiently train deep learning algorithms for postoperative treatment response monitoring.

## 5. Conclusions

In this study, we showed that a state-of-the-art DL algorithm, when trained on the BraTS 2021 training dataset with its current three-label brain tumour annotations, is unsuitable for postoperative segmentation due to the frequent misclassification of resection cavities as tumour regions. To remedy this shortcoming, we automatically converted the annotations of the BraTS 2021 training dataset to a revised two-label annotation protocol adapted for postoperative tumour segmentation. The DL algorithm trained using the two-label annotations accurately excluded resection cavities from tumour volumes while showing a good tumour segmentation performance for larger CE tumours. Our work enables the use of the BraTS 2021 training dataset for training DL algorithms aimed at a postoperative setting and thus equips a broad community of researchers and clinicians with a large body of essential training data to develop algorithms tailored for postoperative brain tumour response assessment, effectively reducing the need for the publicly scarce postoperative training data.

## Figures and Tables

**Figure A1:**
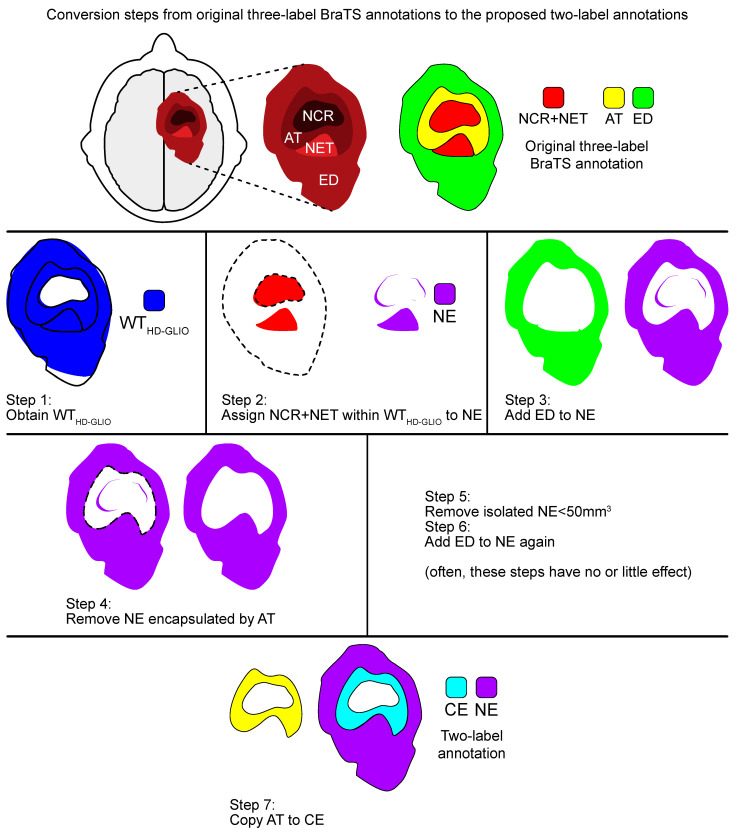
Schematic illustration of the steps of the conversion of the BraTS 2021 three-label segmentation masks for harmonisation with the proposed two-label annotation protocol. The top panel shows the original BraTS three-label annotations and the bottom panel the resulting two-label annotations. At each step, a left illustration shows what is currently treated and how, and a right illustration shows the current result of the process. AT = active contrast-enhancing tumour; ED = oedema and infiltrated tissue; WT_HD-GLIO_ = automatic whole-tumour segmentation by HD-GLIO; CE = contrast-enhancing tumour; NE = non-enhancing hyperintense T2/FLAIR signal abnormalities.

## Data Availability

The script for converting the BraTS three-label annotations to our proposed two-label annotation protocol and the original data generated in the study, i.e., the expert segmentations of the postoperative test dataset, are openly available at GitHub: https://github.com/DEPICT-RH/postoperative_brain_tumor_segmentation_with_brats (accessed on 7 August 2024). The preoperative image data and original three-label annotations used in this study are from the RSNA-ASNR-MICCAI Brain Tumor Segmentation (BraTS) Challenge 2021 and can be requested through: http://braintumorsegmentation.org/ (accessed on 7 August 2024). The postoperative image data and the HD-GLIO segmentation of these are from the public Lumiere dataset, and are available at FigShare: https://springernature.figshare.com/collections/The_LUMIERE_Dataset_Longitudinal_Glioblastoma_MRI_with_Expert_RANO_Evaluation/5904905 (accessed on 7 August 2024).

## Appendix B

The model was trained using nnU-Net [3]. nnU-Net is designed to require minimal optimisation for a new segmentation task, as it automatically extracts hyperparameters during network preprocessing. This is done by automatically defining a set of dataset fingerprints, including image sizes, voxel spacing and class ratios. From this fingerprint, hyperparameters such as the appropriate patch-size, batch-size, number of feature maps and the convolutional kernel size are set.

We utilised the default parameters set after extracting the dataset fingerprints. With the BraTS dataset, this resulted in 1 mm^3^ spacing, a median matrix size of [140,171,136], a patch size of [128,160,112] and a batch size of 2. Image normalisation was performed with z-score normalisation. nnU-Net is publicly available at www.github.com/MIC-DKFZ/nnUNet, (accessed on 7 August 2024). A full description and further details about nnU-Net can be found in [3].

We used the default training setup, which involves training using five-fold cross validation for 1000 epochs, each lasting 250 batches. The training dataset (n = 1000) was randomly split into a training and a test set in each fold prior to training. In our study, we used the ‘3d_fullres’ configuration of nnU-Net.
